# Urapidil alleviates ovarian torsion detorsion injury via regulating oxidative stress, apoptosis, autophagia, and inflammation

**DOI:** 10.22038/ijbms.2021.57196.12736

**Published:** 2021-07

**Authors:** Mustafa Can Güler, Ayhan Tanyeli, Derya Güzel Erdoğan, Ersen Eraslan, Selim Çomaklı, Elif Polat, Songül Doğanay

**Affiliations:** 1 Department of Physiology, Atatürk University, Faculty of Medicine, Erzurum, Turkey; 2 Department of Physiology, Sakarya University, Faculty of Medicine, Sakarya, Turkey; 3 Department of Physiology, Yozgat Bozok University, Faculty of Medicine, Yozgat, Turkey; 4 Department of Pathology, Atatürk University, Veterinary Faculty, Erzurum, Turkey; 5 Department of Nutrition and Dietetics, Faculty of Health Sciences, Erzurum Technical University, Erzurum, Turkey

**Keywords:** Autophagy, Caspase-3, Detorsion, NF-κB, Ovarian, Torsion, Urapidil

## Abstract

**Objective(s):**

This study aimed to determine anti-inflammatory, antioxidant, and antiapoptotic properties of urapidil (Ura) against ovarian torsion detorsion (T/D) injury in rats.

**Materials and Methods:**

40 female Wistar albino rats were grouped as sham, T/D, T/D+dimethyl sulfoxide (DMSO), T/D+Urapidil (Ura) 0.5 mg/kg (low dose), and T/D+Urapidil (Ura) 5 mg/kg (high dose) groups. In treatment groups, Ura was administered intraperitoneally just before detorsion. Biochemical parameters (TAS, TOS, MDA, MPO, and SOD) and immunohistochemical (IL-1β, TNF-α, NF-κB, LC3B, and Caspase-3) analyzes were performed.

**Results:**

In the T/D group, OSI and MPO levels were elevated significantly while TAS values decreased compared with the sham group. A significant difference occurred in the low dose treatment group in TAS and OSI levels compared with the T/D group. In the high dose treatment group, significant elevation in TAS but reduction in OSI and MDA levels were observed compared with the T/D group. Immunohistochemical staining resulted in IL-1β, TNF-α, NF-κB, LC3B, and caspase-3 immunopositivity in the T/D group, while Ura treatment decreased those parameters. Intensive congestion and hemorrhage were observed in the T/D group, but contrary to this, treatment groups had alleviated congestion and hemorrhage.

**Conclusion:**

These results suggest that Ura demonstrated protective effects against ovarian T/D injury via anti-oxidative, anti-inflammatory, and anti-apoptotic features.

## Introduction

Acute ovarian torsion is an emergency characterized by tissue damage. In the clinic, acute ovarian ischemia can be observed due to ovarian torsion, which ranks 5^th^ among gynecological emergencies (1). If there is severe necrosis, oophoropexy might be considered but laparoscopic detorsion is recommended to conserve the ovaries and fertility (2). The main cause of ovarian tissue damage in ovarian torsion is tissue ischemia and subsequent reperfusion injury due to formation of excess reactive oxygen molecules after detorsion, which can be defined as ischemia-reperfusion (I/R) injury (1, 3). Following detorsion, regaining of the blood flow results in reactive oxygen species (ROS) formation. ROS affect biological molecules by clashing with their biological functionality (4, 5). 

ROS induce immune cytotoxic responses in various cell types (6). Oxidative stress stimulates phosphorylated protein activation, including protein kinases that play a key role in cell cycle regulation. Superoxide dismutase (SOD) and several members of the antioxidant system can suppress the cell cycle progression (7, 8). 

During torsion detorsion (T/D), overproduction of ROS primarily leads to tissue damage. Enzymatic and non-enzymatic cellular anti-oxidant mechanisms function synergistically. SOD is produced directly in the intracellular microenvironment and prevents tissues from ROS and oxidative damage. Malondialdehyde (MDA), the final lipid peroxidation product, is produced with ROS and is generally preferred as an indirect indicator of ROS (9). Excessive ROS production may lead to cellular damage resulting in apoptosis of the cell (10). The total antioxidant status (TAS) represents the antioxidative system while the total oxidant status (TOS) demonstrates the oxidative mechanisms (11).

The vasodilator drug urapidil (Ura), the agent that increases tissue oxygen support and has antioxidant effects, was examined in previous studies (12, 13). Ura includes metabolites with antihypertensive activity (14). However, the role of Ura in ovarian T/D injury therapy has not been examined yet. Here, we hypothesized that Ura has protective effects against ovarian tissue injury. 

## Materials and Methods

***Experimental animals and ethical approval***

Atatürk University Experimental Animal Ethics Committee approved the study. Experiments of this study were established at Experimental Animals Research and Application Center (EARAC), Atatürk University (22.12.2017/195). Experimental animals were procured from EARAC. Rats were caged in standard, appropriate laboratory conditions, including 12 hr light/12 hr darkness, 22±2 °C temperature, and humidity of 55±5 %. Standard rat feed and tap water were given to the rats. No feeding was allowed 12 hr before the experiment, but water was available.

***Experimental protocols, drugs, and groups***

Animals were anesthetized using 8 mg/kg xylazine hydrochloride intraperitoneal (IP) (Rompun, Bayer, Turkey) and 75 mg/kg ketamine hydrochloride IP (Ketalar, Eczacıbaşı, Turkey). Following anesthesia, all rats were immobilized in the supine position, and then the abdominal skin was shaved and cleaned. 10% povidone-iodine solution (Batticon; Adeka Laboratories) was used for disinfection. URA was supplied by Sigma-Aldrich Co., USA and had a purity grade of 99%. It was prepared by dissolving in dimethyl sulfoxide (DMSO).

Forty female Wistar albino rats (6–8 months old, 240–270 g) were randomly divided into five groups without resting/training period, in a pathogen-free environment and with 2 rats in each cage. The entire experimental process was carried out under the supervision of a veterinarian appointed by the EARAC.

Group I (Sham, n=8*):* A 1–2-cm sized vertical incision was performed in the abdominal midline (laparotomy) area, but no procedure was carried out. Then, the incision line was closed using a 3/0 silk suture. 

Group II (T/D, n=8): In addition to the laparotomy process in group I, bilateral ovarian arteria and veins, ovaries, and fallopian tubes were spun (torsion) clockwise about 360 degrees and held with microvascular clamps for 3 hr. Then, in the detorsion period, blood circulation was allowed for about 3 hr by opening the clamps (15-18). 

Group III (T/D+DMSO, n=8): In addition to the surgical procedure in group II, 0.3 ml 15% DMSO was applied IP just before detorsion*.*

Group IV (T/D+Ura 0.5 mg/kg, n=8: Same surgical procedure in group II was performed, but a single dose of 0.5 mg/kg Ura was administered IP just prior to detorsion. 

Group V (T/D+Ura 5 mg/kg, n=8): Differently from group IV, a single dose of 5 mg/kg Ura was given IP before the detorsion process start. 

Following detorsion, sacrification of the animals was performed by cardiac puncture, and ovarian tissues were removed.

***Biochemical examination***

10% phosphate buffer solution (PBS) was added to ovarian tissues, and homogenization was performed at 12,000 rpm for 1–2 min on ice (IKA, Germany). The centrifugation process was carried out at +4 °C and 5000 rpm for 30 min to acquire the supernatant, and it was analyzed to determine MDA, TAS, MPO, TOS, and SOD values. 

MDA value was assessed via the method described by Ohkawa *et al.* previously (19). Evaluation of TAS and TOS levels was established with appropriate kits (Rel Assay Diagnostics Ref. No:RL0024 and Rel Assay Diagnostics Ref. No:RL0005, respectively). The ratio of TOS to TAS is known as the oxidative stress index (OSI). Myeloperoxidase (MPO) determination was based on absorbance measurement (20). SOD was gauged following reaction with tetrazolium salt to produce formazan dye (21). 

***Histopathological examination***

The rats were sacrificed after euthanasia, and ovarian tissues were incubated in 10% PBS. Then, a routine follow-up process was applied and fixed in paraffin blocks. Sections of 5 μm taken from the blocks were investigated for histopathological changes. Hematoxylin and Eosin staining was performed, and the sections were investigated using a light microscope. They were considered as none (-), mild (+), moderate (++), or severe (+++) (22).

***Immunohistochemical (IHC) staining***

IHC staining was performed as described in previous studies (23, 24). Tissues were kept in a neutral formaldehyde solution for 24 hr and then washed via drinking water. After passing through alcohol-xylol follow-up, they were put in paraffin blocks. After deparaffinization of the tissues, they were left for 10 min in 3% H_2_O_2_ and washed in phosphate buffer saline (PBS) to prevent peroxidase activity. Then, they were exposed to an antigen retrieval solution at 500w for 10 min. Antigens were removed from tissues by washing in PBS. Cleaved caspase-3 (Novus Biological, Cat. No: NB600-1235, Dilution: 1/100), light chain 3B (LC3B) (Abcam, Cat. No: ab48394 Dilution: 1/200), nuclear factor kappa-B (NF-κB) (Abcam, Cat No: ab7971, Dilution: 1/150), tumor necrosis factor-alpha (TNF-α) (Novus Biological, Cat. No: NBP1-19532, Dilution: 1/100) and interleukin-1beta (IL-1β) (Bioss, Cat. No: bs-0812R, Dilution: 1/100) were applied as the primary antibody. 3,3 ‘diaminobenzidine chromogen was applied, and contrast staining with hematoxylin was done. The samples were analyzed under a light microscope at 20x magnification. Immunopositivity was evaluated as follows: none (−), mild (+), moderate (++), and intense (+++) (25). 

***Statistical analysis***

Statistical analysis was done using SPSS v.20 (IBM, Chicago, USA) software. Nonparametric data were determined using the Kruskal Wallis test. Mann-Whitney U test was used to determine the group that made a difference, and one-way ANOVA was used to evaluate parametric data (26). Tukey’s *post hoc* test was used for multiple comparisons. *P*<0.05 value was considered significant. All data were expressed as the mean±standard error of the mean (SEM). For IHC staining, all data were analyzed with SPSS statistical software (SPSS for Windows, version 20.0). Data are presented as mean±SEM. For IHC analysis, differences were analyzed with a nonparametric test (Kruskal–Wallis) followed by Mann–Whitney U test (*P*<0.05) (25).

## Results

The results were demonstrated in Tables 1 and 2. TAS levels decreased while TOS and OSI levels increased in T/D and TD+DMSO groups compared with the sham group. In Ura treatment groups, TAS levels increased while TOS and OSI levels diminished. No significant difference was observed in MPO activity between groups. While SOD level decreased in T/D and TD+DMSO groups, it was elevated in Ura treatment groups. MDA levels increased in T/D and TD+DMSO groups while decreasing in Ura administered groups.

***Histopathological examination***

**Figure 1:**
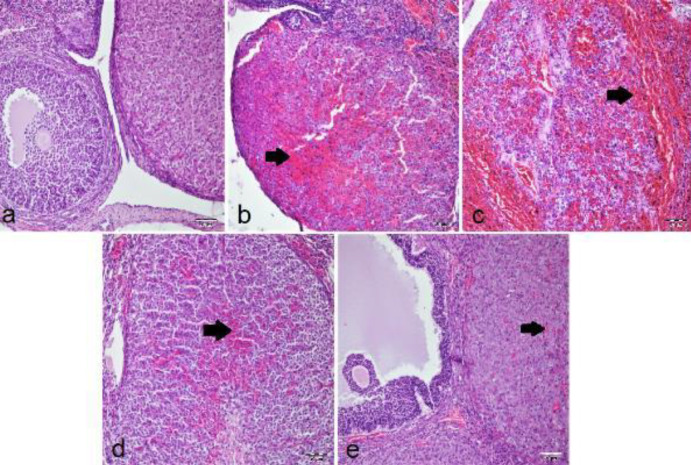
Histopathological images of rat ovarian tissue samples (H&E, ×20 magnification). a) Sham group demonstrated normal ovarian histological structure. b) T/D and c) T/D+DMSO groups’ I/R tissue samples with ovarian sections containing severe hemorrhage (arrows) in rats. d) T/D+Ura 0.5 mg/kg and e) T/D+Ura 5 mg/kg groups’ I/R and Ura-treated samples; ovarian sections containing mild hemorrhage and hyperemia (arrows). DMSO: Dimethyl sulfoxide; Ura: Urapidil; T/D: Torsion detorsion

In the histopathological examination, the ovarian tissues of the sham group had a normal histological appearance (Figure 1a). In other groups, hemorrhages were observed in the interstitial tissue of the corpus luteum. In T/D (Figure 1b) and T/D+DMSO (Figure 1c) groups, it was observed that hemorrhage was intense in both the luteal region and stroma. In T/D+Ura 0.5 mg/kg group (Figure 1d), mild hemorrhage and hyperemia were observed in the stroma. Besides, mild hyperemia was observed in the stroma of the T/D+Ura 5 mg/kg group (Figure 1e).

When T/D and T/D+DMSO groups were evaluated in terms of hemorrhage, parameters were significantly worse than those of the sham and other treatment groups (Table 3). Significantly fewer histopathological changes were found in T/D+Ura 0.5 mg/kg and T/D+Ura 5 mg/kg groups compared with T/D and T/D+DMSO groups. There was no statistical difference between sham and T/D+Ura 5 mg/kg groups (Table 3). 

***IHC examination***

**Figure 2:**
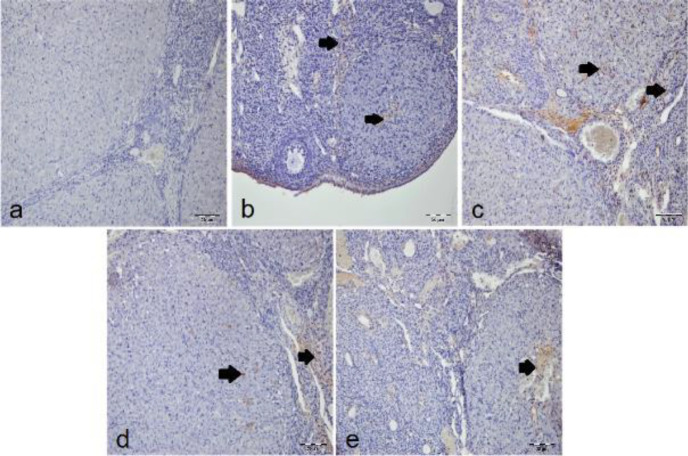
Representative images of the effect of Ura treatment after ovarian T/D injury (x20, IHC): Arrows show IL-1β immune positive cells. a) sham group with negative IL-1β immunopositivity of the lutein and interstitial cells. b) T/D group with intense IL-1β immunopositivity of lutein and interstitial cells. c) T/D+DMSO group with more intense IL-1β immunopositivity of lutein and interstitial cells. d) T/D+Ura 0.5 mg/kg group with moderate IL-1β immunopositivity of lutein and interstitial cells. e) T/D+Ura 5 mg/kg group with mild IL-1β immunopositivity of lutein and interstitial cells

**Figure 3:**
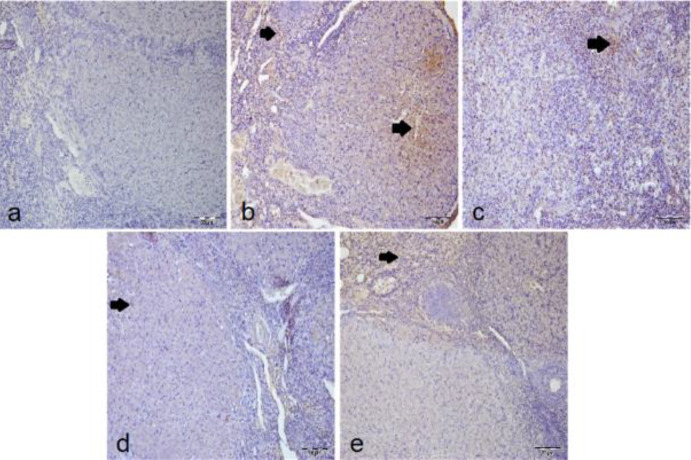
Representative images of the effect of Ura treatment after ovarian T/D injury (x20, IHC): Arrows show TNF-α immune positive cells. a) sham group with negative TNF-α immunopositivity of lutein and interstitial cells. b) T/D group with more intense TNF-α immunopositivity of lutein and interstitial cells. c) T/D+DMSO group with intense TNF-α immunopositivity of the interstitial cells. d) T/D +Ura 0.5 mg/kg group with mild TNF-α immunopositivity of the lutein cells. (e) T/D +Ura 5 mg/kg group with mild IL-1β immunopositivity of the interstitial cells

**Figure 4:**
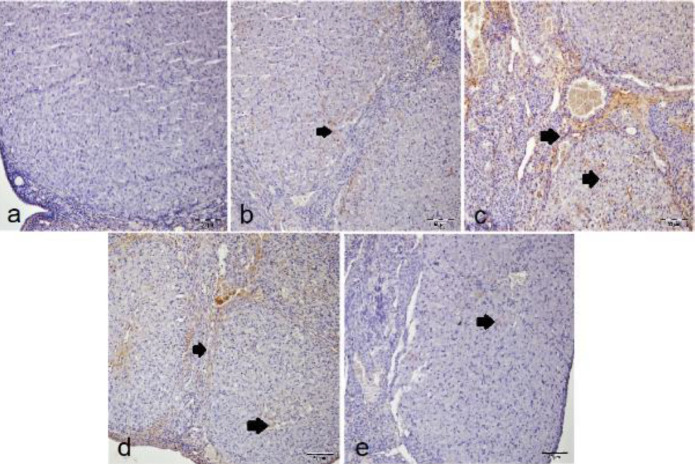
Representative images of the effect of Ura treatment after ovarian T/D injury (x20, IHC): Arrows show NF-κB immune positive cells. a) sham group with negative NF-κB immunopositivity of lutein and interstitial cells. b) T/D group with intense TNF-α immunopositivity of the lutein cells. c) T/D+DMSO group with more intense NF-κB immunopositivity of lutein and interstitial cells. d) T/D +Ura 0.5 mg/kg group with moderate NF-κB immunopositivity of lutein and interstitial cells. e) T/D +Ura 5 mg/kg group with mild NF-κB immunopositivity of the lutein cells

There was no immunopositivity for the IHC investigation of IL-1β, TNF-α, and NF-κB in the sham group (Figures 2a, 3a, and 4a). In the T/D group, intensive immunopositivity was found for these parameters. The most intensive immunopositivity for IL-1β was observed in the T/D+DMSO group (Figure 2c). IL-1β immunopositivity declined in both T/D+Ura 0.5 mg/kg (Figure 2d) and T/D+Ura 5 mg/kg groups (Figure 2e). The IL-1β immunopositivity was significantly higher in the T/D and T/D+DMSO groups than in the sham and other treatment groups (*P*<0.05, Table 3). However, the IL-1β immunopositivity was significantly lower in the T/D+Ura 5 mg/kg group than in the T/D+Ura 5 mg/kg group (*P*<0.05, Table 3). There was no statistical difference between the sham and T/D+Ura 5 mg/kg groups (*P*>0.05, Table 3).

In the T/D+DMSO group (Figure 3c), TNF-α immunopositivity occurred intensively, but the most intensive immunopositivity was observed in the T/D group (Figure 3b). In treatment groups (Figures 3d and e), TNF-α immunopositivity decreased. TNF-α immunopositivity of the sham group was significantly lower than the other groups (*P*<0.05, Table 3). TNF-α immunopositivity increased in the T/D and T/D+DMSO groups (*P*<0.05, Table 3) and decreased in the T/D+Ura 0.5 mg/kg and T/D+Ura 5 mg/kg groups (*P*<0.05, Table 3). There was no statistical difference between the T/D+Ura 0.5 mg/kg and T/D+Ura 5 mg/kg groups (*P*>0.05, Table 3).

NF-κB immunopositivity was most intense in the T/D+DMSO group (Figure 4c). In T/D (Figure 4b) and T/D+Ura 0.5 mg/kg (Figure 4d) groups, NF-κB immunopositivity was intense, while in T/D+Ura 5 mg/kg group (Figure 4e), NF-κB immunopositivity diminished. Increased NF-kB immunopositivity was observed in T/D and T/D+DMSO groups compared with sham and T/D+Ura 5 mg/kg groups (*P*<0.05, Table 3). Decreased NF-kB immunopositivity was observed in the T/D+Ura groups, but this decrease only reached a statistical significance in the T/D+Ura 5 mg/kg group (*P*<0.05, Table 3).

**Figure 5:**
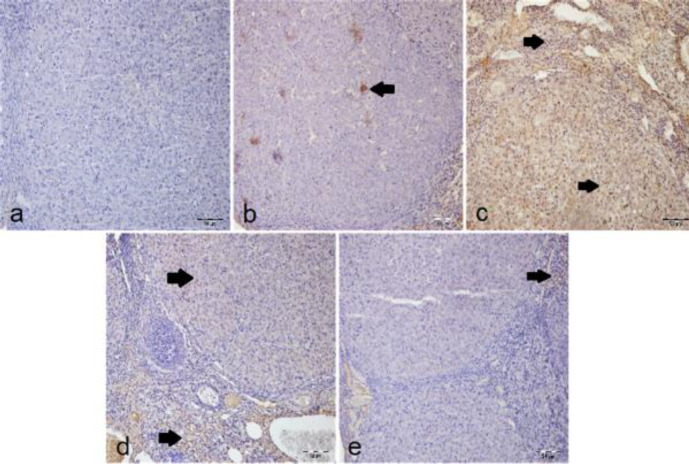
Representative images of the effect of Ura treatment after ovarian T/D injury (x20, IHC): Arrows show caspase-3 immune positive cells. a) sham group with negative caspase-3 immunopositivity of the lutein cells. b) T/D group with intense caspase-3 immunopositivity of the lutein cells. c) T/D+DMSO group with more intense caspase-3 immunopositivity of lutein and interstitial cells. d) T/D +Ura 0.5 mg/kg group with intense caspase-3 immunopositivity of lutein and interstitial cells. e) T/D +Ura 5 mg/kg group with mild caspase-3 immunopositivity of the interstitial cells

There was no caspase-3 immunopositivity in the sham group (Figure 5a). The most intense caspase-3 immunopositivity was seen in the T/D+DMSO group (Figure 5c). In the T/D group (Figure 5b), caspase-3 immunopositivity was less than in the T/D+DMSO group. Besides, caspase-3 immunopositivity was mild in the T/D+Ura 0.5 mg/kg group (Figure 5d), and the lightest immunopositivity occurred in T/D+Ura 5 mg/kg group (Figure 5e). When the groups were examined, it was shown that the caspase-3 immunopositivity did not occur in the sham group (*P*<0.05, Table 3). Caspase 3 immunopositivity in the T/D+Ura 5 mg/kg group was significantly lower compared with those in T/D, T/D+DMSO and T/D+Ura 0.5 mg/kg groups (*P*<0.05, Table 3). No statistically significant difference was identified between sham and T/D+Ura 5 mg/kg groups in terms of caspase 3 immunopositivity (*P*>0.05, Table 3).

**Figure 6:**
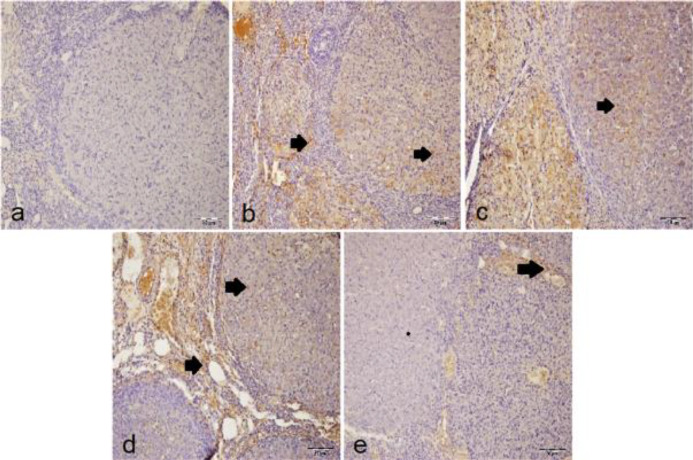
Representative images of the effect of UR treatment after ovarian torsion detorsion injury (x20, IHC): Arrows show LC3B immune positive cells. (a) sham group with negative LC3B immunopositivity of lutein and interstitial cells. (b) T/D group with intense LC3B immunopositivity of lutein and interstitial cells. (c) T/D+DMSO group with intense LC3B immunopositivity of the lutein cells. (d) T/D +Ura 0.5 mg/kg group with intense LC3B immunopositivity of lutein and interstitial cells. (e) T/D +Ura 5 mg/kg group with mild IL-1β immunopositivity of the interstitial cells

In IHC staining in terms of autophagic cell death, no immune positivity was found in the sham group for LC3B (Figure 6a). In T/D (Figure 6b), T/D+DMSO (Figure 6c), and T/D+Ura 0.5 mg/kg groups (Figure 6d), LC3B immunopositivity was intense, while in T/D+Ura 5 mg/kg group (Figure 6e), it was mild. When the groups were examined, it was shown that LC3B immunopositivity in the sham group was not found (*P*<0.05, Table 3). LC3B immunopositivity in the T/D+Ura 5 mg/kg group was significantly lower compared with those in the T/D, T/D+DMSO, and T/D+Ura 0.5 mg/kg groups (*P*<0.05, Table 3). No statistically significant difference was identified between the T/D, T/D+DMSO, and T/D+Ura 0.5 mg/kg groups in terms of LC3B immunopositivity (*P*>0.05, Table 3).

**Table 1:** Comparison of TAS, TOS, and OSI parameters between the experimental groups

**Experimental Groups (n=8) **	**TAS ** **(mmol/L)**	**TOS ** **(µmol/L)**	**OSI (arbitrary unit)**
**Sham **	0,74 ± 0,22	5,03 ± 0,41	0,73 ± 0,23
**T** **/D**	0,26 ± 0,16^a^	9,95 ± 1,63^a^	5,24 ± 2,82^a^
**T** **/D** **+DMSO **	0,25 ± 0,05^a^	9,51 ± 0,50^a^	3,80 ± 0,76^a^
**T/D+Ura 0,5 mg/kg **	0,56 ± 0,12^b^	6,41 ± 0,70^b^	1,17 ± 0,23^b^
**T/D+Ura 5 mg/kg **	0,56 ± 0,07^b^	5,81 ± 0,69^b^	0,98 ± 0,25^b^

a*P*<0.001 and c*P*<0.05 compared with sham group. b*P*<0.001 compared with T/D group and T/D+DMSO group

TAS: Total anti-oxidant status; TOS: Total oxidant status; OSI: Oxidative stress index; DMSO: Dimethyl sulfoxide; Ura: Urapidil

**Table 2:** Comparison of SOD, MPO, and MDA values between the experimental groups

**Experimental Groups (n=8) **	**SOD ** **(U/mg protein)**	**MPO ** **(U/g protein)**	**MDA ** **(µmol/g protein)**
**Sham **	382,92 ± 128,72	329571,29 ± 270573,09	67,86 ± 6,67
**T** **/D**	157,67 ± 43,03^a^	445780,11 ± 274801,48	97,03 ± 25,61^c^
**T** **/D** **+DMSO **	154,36 ± 27,80^a^	440190,20 ± 127341,80	91,48 ± 14,62^c^
**T/D+Ura 0,5 mg/kg **	340,11 ± 116,92^b^	186335,16 ± 45318,83	75,90 ± 4,55^b^
**T/D+Ura 5 mg/kg **	362,22 ± 73,77^b^	289,041 ± 49798,44	71,39 ± 7,88^b^

a*P*<0.001 and c*P*<0.05 compared with sham group. b*P*<0.001 compared with T/D group and T/D+DMSO group

SOD: Superoxide dismutase; MPO: Myeloperoxidase; MDA: Malondialdehyde; DMSO: Dimethyl sulfoxide; Ura: Urapidil

**Table 3:** Histopathological score and immunopositivity evaluation of the experimental groups

**Groups**	**Histopathological ** **score**	**IL-1β**	**TNF-α**	**NF-kB**	**Caspase-3**	**LC3B**
**Sham**	0.12±0.12^a^	0.25±0.16^a^	0.12±0.12^a^	0.37±0.26^a^	0.00±0.00^a^	0.12±0.12^a^
**T/D**	1.87±0.22^b^	2.12±0.39^b^	2.75±0.16^b^	2.37±0.18^b^	1.75±0.31^b^	2.12±0.22^b^
**T/D+DMSO**	2.50±0.26^b^	2.62±0.18^b^	2.25±0.25^b^	2.87±0.12^c^	2.87±0.12^c^	2.62±0.18^b^
**T/D+Ura 0.5 mg/kg**	1.25±0.31^c^	1.50±0.26^c^	1.25±0.31^c^	1.87±0.29^b^	1.12±0.35^b^	1.87±0.35^b^
**T/D+Ura 5 mg/kg**	0.75±0.25^a^	1.12±0.29^a^	1.25±0.25^c^	1.12±0.22^d^	0.62±0.26^a^	0.50±0.18^c^

All data were expressed as mean±SEM. Different superscript letters (a, b, c, d) indicate statistical differences between the groups

IL-1β: Interleukin-1 beta; TNF-α: Tumor necrosis factor-alpha; NF-kB: Nuclear factor-kappa B; LC3B: Light chain 3B; DMSO: Dimethyl sulfoxide; Ura: Urapidil; T/D: Torsion detorsion

Ura: Urapidil; T/D: Torsion detorsion

Ura: Urapidil; T/D: Torsion detorsion

Ura: Urapidil; T/D: Torsion detorsion

Ura: Urapidil; T/D: Torsion detorsion; DMSO: Dimethyl sulfoxide

Ura: Urapidil; T/D: Torsion detorsion

## Discussion

Ovarian torsion is mostly observed during the first 3 decades of life (27). In adolescence, conditions such as excessive tube mobility and overgrowth of the ovaries play a role in the etiology (28). Ischemia duration is important. It has been reported yhat it is possible to return the adnexa to the previous condition within the 18–14 hr following the ischemia (29). Besides, unilateral torsion affects the contralateral ovary in terms of ovulation (30, 31). In a previous study, several changes were observed in the contralateral ovaries following ipsilateral ovarian ischemia. This may result from the sympathetic system stimulation by the unilateral ovarian ischemia which lowers the blood flow as in testicular torsion (31). 

Ura declines the peripheral vascular resistance acting as an α1-adrenoceptor antagonist (32). By examining the literature, we can list the healing effects of Ura in this study as follows: First; abnormal catecholamine secretion with complications including myocardial infarction, cardiac failure, and other I/R damage is a major challenge for the treatment management. Ura is a useful and cost-effective choice in the treatment method of patients for example pheochromocytoma perioperative treatment (33). Second; epinephrine and norepinephrine play a role in iron release from ferritin during I/R. This means catecholamines perform a negative activity in the presence of free radicals (34). Catecholamine-iron complexes generate reactive radicals through lipid peroxidases (35). Ura may decline iron ion release from ferritin that may be a potential mechanism of α1-adrenoceptor blocking. 

Blood via excessive oxygen amounts during the reperfusion phase leads to neutrophil and ROS formation, damaging the tissue (36). Reperfusion is necessary for oxygen and supplement support but injures tissues via ROS generation and apoptosis (37). High levels of oxygen reach the tissues which leads to excessive ROS formation during the detorsion phase. In our study, TOS, which shows the total value of oxidative stress, and OSI, which shows the balance between antioxidants and oxidants, increased in the study groups in favor of oxidative damage, and these effects reversely changed with Ura treatment. ROS damage DNA and induce lipid peroxidation (38, 39). Lipid peroxidation is quite harmful to the cell, which results from free radicals. MDA leads to damage in the cell by enhancing polymerization and cross-linking membrane components (40). In our study, MDA increased due to the damage that occurred in the study groups and decreased with Ura treatment. Antioxidant body mechanisms scavenge ROS and protect tissues. If oxidant mechanisms override the antioxidant systems, this results in increased ROS formation, lipid peroxidation, and tissue injury (41). MPO activity may increase due to I/R injury in ovarian tissues (42). MPO activity indicates neutrophil activation, which is related to ROS generation and cytokine production (43). IL-2, IL-6, IL-1β, and TNF-α are proinflammatory cytokines produced during I/R injury (44, 45). IL-1β enhances inflammation and apoptosis rate (46, 47). TNF-α is well known as one of the key cytokines mediating inflammatory responses (48). I/R injury enhances inflammation by releasing factors including IL-6, TNF-α, and IL-8. These factors lead to organ damage by playing a role in inflammation (49, 50). NF-κB enhances the activation of the various proinflammatory cytokines such as TNF-α, IL-6, COX-2, and IL-1β (51). Reperfusion-related tissue injury mostly depends on IL-1β and TNF-α (52, 53). In this study, IL-1β and TNF-α levels declined due to Ura treatment which supports the decrease of inflammation.

SOD, GPx, and catalase (CAT) prevent the undesirable effects of ROS (54). OSI, the rate of TOS to TAS (55), is a sensitive indicator for oxidative stress assessment (56). ROS generation in the reperfusion stage mainly acts on uncontrolled oxidative stress, and high ROS levels play a role in the inflammatory cascade (57). ROS generation is associated with proapoptotic gene regulation, caspase activation, and apoptosis-related cell death (58). If antioxidants cannot suppress ROS, it results in ovarian damage (59). 

Autophagy, cell death, and cell proliferation are related to each other (60). Constantine autophagy modulates apoptosis, and thus, autophagy is a programmed cell death (61). Apoptosis is a self-killing function, and cells with apoptosis are excreted in a controlled manner (62). The caspases also play important roles in the regulation of autophagy, apart from their role in apoptosis. They are activated in response to autophagy (63). Caspase-3 acts as a common effector caspase in all three apoptotic pathways (64). LC3B and caspase-3 expressions demonstrate an increase in various ischemia models (65, 66). 

In the current study, it was determined that autophagy and apoptosis were triggered during ovarian ischemia. It was also shown that the administration of different doses of Ura suppresses the level of autophagosome marker LC3B and caspase-3. Decreased apoptosis and autophagy following ischemic damage in Ura groups are associated with autophagosome marker LC3B. 

## Conclusion

Our study showed that Ura can elevate SOD and TAS activity, and reduce the TOS level significantly in ovarian tissue injury. Histopathological analysis showed that Ura reduced inflammatory cytokines, apoptosis, and autophagy with rising doses. In conclusion, Ura has a protective effect against ovarian T/D injury in rats.

## References

[1] Cenk Y, Dural Ö, baştu E, uğurlucan FG, Fatih K, İyi̇bozkurt AC (2016). Over torsi̇yonu olgusunda konservati̇f yaklaşimin over rezervi̇ üzeri̇ne etki̇si̇ni̇n anti̇mülleri̇yan hormon düzeyi̇ ve antral foli̇kül sayisi i̇le değerlendi̇ri̇lmesi̇: Olgu sunumu. İstanbul Tıp Fakültesi Dergisi.

[2] Laganà AS, Sofo V, Salmeri FM, Palmara VI, Triolo O, Terzić MM (2016). Oxidative stress during ovarian torsion in pediatric and adolescent patients: Changing the perspective of the disease. Int J Fertil Steril.

[3] Sengul O, Ferah I, Polat B, Halici Z, Bayir Y, Yilmaz M (2013). Blockade of endothelin receptors with bosentan limits ischaemia/reperfusion-induced injury in rat ovaries. Eur J Obstet Gynecol Reprod Biol.

[4] Meister A (1988). Glutathione metabolism and its selective modification. J Biol Chem.

[5] Kultz D (2005). Molecular and evolutionary basis of the cellular stress response. Annu Rev Physiol.

[6] Paravicini TM, Touyz RM (2008). NADPH oxidases, reactive oxygen species, and hypertension: clinical implications and therapeutic possibilities. Diabetes Care.

[7] Kara M, Daglioglu YK, Kuyucu Y, Tuli A, Tap O (2012). The effect of edaravone on ischemia–reperfusion injury in rat ovary. Eur J Obstet Gynecol Reprod Biol.

[8] Preston TJ, Muller WJ, Singh G (2001). Scavenging of extracellular H2O2 by catalase inhibits the proliferation of HER-2/Neu-transformed rat-1 fibroblasts through the induction of a stress response. J Biol Chem.

[9] Meštrović J, Pogorelić Z, Drmić-Hofman I, Vilović K, Todorić D, Popović M (2017). Protective effect of urapidil on testicular torsion–detorsion injury in rats. Surgery Today.

[10] Lee JY, Baw C-K, Gupta S, Aziz N, Agarwal A (2010). role of oxidative stress in polycystic ovary syndrome Curr. Women's Health Rev.

[11] Erel O (2004). A novel automated method to measure total antioxidant response against potent free radical reactions. Clin Biochem.

[12] Higginson L, Tang A, Knoll G, Calvin J (1991). Effect of intracoronary diltiazem on infarct size and regional myocardial function in the ischemic reperfused canine heart. J Am Coll Cardiol.

[13] Meštrović J, Drmić-Hofman I, Pogorelić Z, Vilović K, Šupe-Domić D, Šešelja-Perišin A (2014). Beneficial effect of nifedipine on testicular torsion-detorsion injury in rats. Urology.

[14] Kirsten R, Nelson K, Steinijans VW, Zech K, Haerlin R (1988). Clinical pharmacokinetics of urapidil. Clin Pharmacokinet.

[15] Güler MC, Tanyeli A, Eraslan E, Ekinci Akdemir FN (2020). Role of 6-shogaol against ovarian torsion detorsion-induced reproductive organ damage. NTMS.

[16] Güler MC, Tanyeli A (2020). Role of hyperoside on ovarian tissue damage created by ovarian torsion detorsion. New Trends in Medicine Sciences.

[17] Hascalik S, Celik O, Turkoz Y, Hascalik M, Cigremis Y, Mizrak B (2004). Resveratrol, a red wine constituent polyphenol, protects from ischemia-reperfusion damage of the ovaries. Gynecol Obstet Invest.

[18] Celik O, Turkoz Y, Hascalik S, Hascalik M, Cigremis Y, Mizrak B (2004). The protective effect of caffeic acid phenethyl ester on ischemia-reperfusion injury in rat ovary. Eur J Obstet Gynecol Reprod Biol.

[19] Ohkawa H, Ohishi N, Yagi K (1979). Assay for lipid peroxides in animal tissues by thiobarbituric acid reaction. Analytical biochemistry.

[20] Bradley PP, Priebat DA, Christensen RD, Rothstein G (1982). Measurement of cutaneous inflammation: Estimation of neutrophil content with an enzyme marker. J Invest Dermatol.

[21] Sun Y, Oberley LW, Li Y (1988). A simple method for clinical assay of superoxide dismutase. Clin Chem.

[22] Küçükler S, Çomaklı S, Özdemir S, Çağlayan C, Kandemir FM (2021). Hesperidin protects against the chlorpyrifos-induced chronic hepato-renal toxicity in rats associated with oxidative stress, inflammation, apoptosis, autophagy, and up-regulation of PARP-1/VEGF. Environ Toxicol.

[23] Topdağı Ö, Tanyeli A, Akdemir FNE, Eraslan E, Güler MC, Çomaklı S (2020). Preventive effects of fraxin on ischemia/reperfusion-induced acute kidney injury in rats. Life Sci.

[24] Özdemir S, Kucukler S, Çomaklı S, Kandemir FM (2020). The protective effect of Morin against ifosfamide-induced acute liver injury in rats associated with the inhibition of DNA damage and apoptosis. Drug Chem Toxicol.

[25] Çelik H, Kandemir FM, Caglayan C, Özdemir S, Çomaklı S, Kucukler S (2020). Neuroprotective effect of rutin against colistin-induced oxidative stress, inflammation and apoptosis in rat brain associated with the CREB/BDNF expressions. Mol Biol Rep.

[26] Topdagi O, Tanyeli A, Akdemir FNE, Eraslan E, Guler MC, Comakli S (2020). Preventive effects of fraxin on ischemia/reperfusion-induced acute kidney injury in rats. Life Sci.

[27] McWilliams GD, Hill MJ, Dietrich CS, 3rd (2008). Gynecologic emergencies. Surg Clin North Am.

[28] Rousseau V, Massicot R, Darwish AA, Sauvat F, Emond S, Thibaud E (2008). Emergency management and conservative surgery of ovarian torsion in children: a report of 40 cases. J Pediatr Adolesc Gynecol.

[29] Macdougall IC (2008). Novel erythropoiesis-stimulating agents: A new era in anemia management. Clin J Am Soc Nephrol.

[30] Coleman DA, Fleming MW, Dailey RA (1984). Factors affecting ovarian compensation after unilateral ovariectomy in gilts. J Anim Sci.

[31] Akgur FM, Kilinc K, Tanyel FC, Buyukpamukcu N, Hicsonmez A (1994). Ipsilateral and contralateral testicular biochemical acute changes after unilateral testicular torsion and detorsion. Urology.

[32] Langtry HD, Mammen GJ, Sorkin EM (1989). Urapidil. A review of its pharmacodynamic and pharmacokinetic properties, and therapeutic potential in the treatment of hypertension. Drugs.

[33] Habbe N, Ruger F, Bojunga J, Bechstein WO, Holzer K (2013). Urapidil in the preoperative treatment of pheochromocytomas: a safe and cost-effective method. World J Surg.

[34] Eckler K, Laufer MR, Perlman SE (2000). Conservative management of bilateral asynchronous adnexal torsion with necrosis in a prepubescent girl. J Pediatr Surg.

[35] Webb EM, Green GE, Scoutt LM (2004). Adnexal mass with pelvic pain. Radiol Clin North Am.

[36] Zimmerman BJ, Granger DN (1992). Reperfusion injury. Surg Clin North Am.

[37] Jassem W, Heaton ND (2004). The role of mitochondria in ischemia/reperfusion injury in organ transplantation. Kidney Int.

[38] Li C, Jackson RM (2002). Reactive species mechanisms of cellular hypoxia-reoxygenation injury. Am J Physiol Cell Physiol.

[39] Grace PA (1994). Ischaemia-reperfusion injury. Br J Surg.

[40] Girotti AW (1998). Lipid hydroperoxide generation, turnover, and effector action in biological systems. J Lipid Res.

[41] Lee DM, Hoffman WH, Carl GF, Khichi M, Cornwell PE (2002). Lipid peroxidation and anti-oxidant vitamins prior to, during, and after correction of diabetic ketoacidosis. J Diabetes Complications.

[42] Meister A (1991). Glutathione deficiency produced by inhibition of its synthesis, and its reversal; applications in research and therapy. Pharmacol Ther.

[43] Wang L, Shan Y, Chen L, Lin B, Xiong X, Lin L (2016). Colchicine protects rat skeletal muscle from ischemia/reperfusion injury by suppressing oxidative stress and inflammation. Iran J Basic Med Sci.

[44] Munoz M, Lopez-Oliva ME, Pinilla E, Martinez MP, Sanchez A, Rodriguez C (2017). CYP epoxygenase-derived H2O2 is involved in the endothelium-derived hyperpolarization (EDH) and relaxation of intrarenal arteries. Free Radic Biol Med.

[45] Huang R, Zhou Q, Veeraragoo P, Yu H, Xiao Z (2011). Notch2/Hes-1 pathway plays an important role in renal ischemia and reperfusion injury-associated inflammation and apoptosis and the gamma-secretase inhibitor DAPT has a nephroprotective effect. Ren Fail.

[46] Eltzschig HK, Collard CD (2004). Vascular ischaemia and reperfusion injury. Br Med Bull.

[47] Hasturk A, Atalay B, Calisaneller T, Ozdemir O, Oruckaptan H, Altinors N (2009). Analysis of serum pro-inflammatory cytokine levels after rat spinal cord ischemia/reperfusion injury and correlation with tissue damage. Turk Neurosurg.

[48] Takada M, Nadeau KC, Shaw GD, Marquette KA, Tilney NL (1997). The cytokine-adhesion molecule cascade in ischemia/reperfusion injury of the rat kidney. Inhibition by a soluble P-selectin ligand. J Clin Invest.

[49] Hashmp SF, Sattar MZA, Rathore HA, Ahmadi A, Johns EJ (2017). A critical review on pharmacological significance of hydrogen sulfide (h(2)s) on nf-kappab concentration and icam-1 expression in renal ischemia reperfusion injury. Acta Pol Pharm.

[50] Peng J, Ren X, Lan T, Chen Y, Shao Z, Yang C (2016). Renoprotective effects of ursolic acid on ischemia/reperfusioninduced acute kidney injury through oxidative stress, inflammation and the inhibition of STAT3 and NFkappaB activities. Mol Med Rep.

[51] Tao Y, Chen YC, Lan T, Qian H, Wang Y, Jiang L (2012). LPS-induced nuclear translocation of RhoA is dependent on NF-kappaB in the human lung cancer cell line A549. Oncol Lett.

[52] Guzman-de la Garza FJ, Ibarra-Hernandez JM, Cordero-Perez P, Villegas-Quintero P, Villarreal-Ovalle CI, Torres-Gonzalez L (2013). Temporal relationship of serum markers and tissue damage during acute intestinal ischemia/reperfusion. Clinics (Sao Paulo).

[53] Yang Q, Zheng FP, Zhan YS, Tao J, Tan SW, Liu HL (2013). Tumor necrosis factor-alpha mediates JNK activation response to intestinal ischemia-reperfusion injury. World J Gastroenterol.

[54] Kusano C, Ferrari C (2008). Total antioxidant capacity: A Biomarker in Biomedical and and Nutrirional Studies. J Mol Cell Biol.

[55] Keith ES, Powers JJ (1965). Effect of phenolic acids and esters on respiration and reproduction of bacteria in urine. Appl Microbiol.

[56] Erel O (2005). A new automated colorimetric method for measuring total oxidant status. Clin Biochem.

[57] Wang L, Liu X, Chen H, Chen Z, Weng X, Qiu T (2015). Effect of picroside II on apoptosis induced by renal ischemia/reperfusion injury in rats. Exp Ther Med.

[58] Tuncer AA, Bozkurt MF, Koken T, Dogan N, Pektas MK, Baskin Embleton D (2016). The protective effects of Alpha-lipoic acid and coenzyme Q10 combination on ovarian ischemia-reperfusion injury: An experimental study. Adv Med.

[59] Bozkurt S, Arikan DC, Kurutas EB, Sayar H, Okumus M, Coskun A (2012). Selenium has a protective effect on ischemia/reperfusion injury in a rat ovary model: Biochemical and histopathologic evaluation. J Pediatr Surg.

[60] Dong Y, Chen H, Gao J, Liu Y, Li J, Wang J (2019). Molecular machinery and interplay of apoptosis and autophagy in coronary heart disease. J Mol Cell Cardiol.

[61] Fuchs Y, Steller H (2011). Programmed cell death in animal development and disease. Cell.

[62] Chen W, Sun Y, Liu K, Sun X (2014). Autophagy: A double-edged sword for neuronal survival after cerebral ischemia. Neural Regen Res.

[63] Song S, Tan J, Miao Y, Li M, Zhang Q (2017). Crosstalk of autophagy and apoptosis: Involvement of the dual role of autophagy under ER stress. J Cell Physiol.

[64] Yagami T, Yamamoto Y, Koma H (2019). Pathophysiological roles of intracellular proteases in neuronal development and neurological diseases. Mol Neurobiol.

[65] Matsui Y, Takagi H, Qu X, Abdellatif M, Sakoda H, Asano T (2007). Distinct roles of autophagy in the heart during ischemia and reperfusion: Roles of AMP-activated protein kinase and Beclin 1 in mediating autophagy. Circ Res.

[66] Ozturk D, Tanyeli A, Çomaklı S, Baylan H, Polat E (2019). The protective effects of urapidil on lung issue after intestinal ischemia-reperfusion injury. Turkish J Biochem.

